# Comparison of first versus second line sacrocolpopexies in terms of morbidity and mid-term efficacy

**DOI:** 10.1038/s41598-022-20127-5

**Published:** 2022-09-29

**Authors:** Marine Lallemant, A. T. M. Grob, M. Puyraveau, M. A. G. Perik, A. H. H. Alhafidh, M. Cosson, R. Ramanah

**Affiliations:** 1 Department of Gynecologic Surgery, Besancon University Medical Centre, 3 Alexander Fleming Boulevard, 25000 Besançon, France; 2 Department of Applied Mechanics, FEMTO-ST Institute, University of Franche-Comte, UMR 6174 CNRS, Besançon, France; 3 Multi-Modality Medical Imaging, Faculty of Science and Technology, Technical Medical Centre, University of Twente, Enschede, The Netherlands; 4 Department of Obstetrics and Gynaecology, Ziekenhuisgroep Twente, Almelo, The Netherlands; 5 Methodology Department, uMETh, Inserm CIC 1431, Besancon University Medical Centre, Besançon, France; 6 Department of Gynecologic Surgery, Jeanne de Flandre, University Medical Centre, Lille, France; 7 Nanomedicine Imaging and Therapeutics Laboratory, INSERM EA 4662, University of Franche-Comte, Besançon, France

**Keywords:** Medical research, Urology, Urogenital diseases

## Abstract

To compare pelvic organ prolapse (POP) recurrence and morbidity between first and second line sacrocolpopexies. We conducted a retrospective chart review of all laparoscopic or robotic sacrocolpopexies for POP-Q stage** ≥ **2, with or without a history of previous prolapse repair, performed with a similar technique between January 2012 and June 2019 in 3 European Gynecologic Surgery Departments. Patients were separated into two groups: first line sacrocolpopexy (FLS) and second line sacrocolpopexy (SLS). Each patient from the SLS group was age-matched with a patient from the FLS group. The primary outcome measure was reoperation procedures for recurrent POP defined as a symptomatic POP-Q stage ≥ 2 POP in at least one vaginal compartment. Secondary outcomes included operative time, intraoperative organ trauma, intraoperative blood loss, postoperative POP recurrence (operated on or not), global reoperation and mesh-related complications. During this period, 332 patients were included. After age-matching, 170 patients were analyzed: 85 patients in the FLS and SLS groups, respectively. After a mean follow-up of 3 years, there was no statistically significant difference between the two groups in terms of recurrent POP (9.4% versus 10.6%, p = 0.7), recurrent POP reoperation (3.5% versus 5.9% p = 0.7), mesh-related reoperation (0% versus 2.4%, p = 0.5), global reoperation (3.5 versus 8.2%, p = 0.3), operative time (198 ± 67 min versus 193 ± 60 min, p = 0.5), intraoperative complications such as organ injury (4.7% versus 7.1%, p = 0.7) and blood loss > 500 mL (2.4% versus 0%, p = 0.5). Patients who underwent a first or a second line sacrocolpopexy seemed to have similar rates of prolapse recurrence and complications.

## Introduction

In Europe there is a disparity in the surgical management of pelvic organ prolapse (POP)^1^. According to Haya et al., sacrocolpopexy for apical prolapse was employed 13 times more frequently in France (66%) than in Sweden (5%)^2^. In France, minimally invasive sacrocolpopexy is usually proposed as a first-line treatment after evaluation of the risk–benefit ratio of performing an abdominal surgery^3,4^. On the contrary, in the Netherlands, sacrocolpopexy is performed as a second line procedure after failure of a previous vaginal POP surgery (e.g. sacrospinous ligament fixation, colporrhaphy, the Manchester Fothergill procedure)^5^. Sacrocolpopexy is a surgery performed for pelvic organ prolapse (POP) with satisfactory results^6^. The anatomical success rate for minimally invasive sacrocolpopexy varies between 77 and 96% on a follow-up of 1–5 years^6–12^. According to Maher et al. meta-analysis^13^, abdominal sacrocolpopexy is associated with a lower risk of subjective failure, a lower rate of recurrent vault prolapse as well as less urinary stress incontinence and dyspareunia, compared to vaginal sacrospinous colpopexy. On the other hand, the operative time procedure is longer as compared to other prolapse surgeries^14^. It is a more expensive surgery^15^ that uses mesh which entails specific costs and complication such as mesh exposure, pain and infection^13,16,17^.

In the literature, there is no study comparing sacrocolpopexy as either first- or second-line surgical management of POP. Therefore, we hypothesize that there is no significant difference in terms of POP recurrence between sacrocolpopexy performed as first line treatment in patients without previous prolapse surgery and sacrocolpopexy performed as second line treatment (prolapse recurrence) in patients with a history of vaginal prolapse surgery. Additionally, a higher morbidity defined by perioperative complications is assumed in patients with sacrocolpopexy as second line treatment due to difficult dissection.

The aim of this study was to compare POP recurrence and morbidity between first and second line sacrocolpopexies.

## Methods

We conducted a retrospective chart review of all minimally invasive sacrocolpopexies performed between January 2012 and June 2019 in 3 European Gynecologic Surgery Departments: Besancon University Medical Center (France), Lille University Medical Center (France) and Ziekenhuisgroep Twente in Almelo (The Netherlands). The study protocol was approved by the Institutional Review Board of the French College of Obstetricians and Gynecologists (CEROG 2019-GYN-0203) and by the Dutch local Institutional Review Board (no. 088-708 34 87). All methods were performed in accordance with the relevant guidelines and regulations. Informed consent was obtained from all participants.

Inclusion criteria were all patients ≥ 18 years old who underwent a laparoscopic or robotic-assisted sacrocolpopexy for POP repair with a POP-Q (Pelvic Organ Prolapse Quantification) stage ≥ 2 with or without a history of previous prolapse repair. POP surgeries were only performed if patients were symptomatic. Only patients with a similar surgical technique according to the operative reports were included: dissection of the vagina anteriorly towards the bladder neck only, fixation on the levator ani muscle only if concerned and mesh attachment on the promontory with knots. Exclusion criteria were sacrohysteropexy, scheduled sacrocolpopexy by laparotomy, concomitant rectopexy or fixation of the mesh on the vesical dome anteriorly.

Patients were separated into two groups: first line sacrocolpopexy (FLS) and second line sacrocolpopexy (SLS). Each patient from the SLS group was age-matched with a patient from the FLS group (age groups used < 40, 40–49, 50–59, 60–69, 70–79, > 80) in a ratio of 1:1.

After induction of general anesthesia, the patient was positioned in dorsal lithotomy position associated with Trendelenburg position. Trocars were inserted after creating a pneumoperitoneum using the Veress needle method or an open technique (Hasson).

All robotic procedures were performed using a robotic four-arm Da-Vinci surgical system (Intuitive Surgical Inc., Sunnyvale, California, USA). Trocars were inserted under visual control: one 12-mm umbilical port for the laparoscope, three 8-mm robotic ports on the same horizontal line or one in each iliac fossa and one in the left flank and one 10-mm conventional laparoscopic port in the right hypochondrium for the assistant surgeon.

During the laparoscopic approach, a 10-mm umbilical trocar was placed for the laparoscope. Two 5-mm right and left iliac trocars and one 10-mm suprapubic trocar were inserted. The surgeon was installed on the left side of the patient and his first assistant on the right. Knots were performed extracorporeally or intracorporeally according to the surgeon's preference.

Regardless of the surgical approach, the sacrocolpopexy was performed as previously described by Cosson et al.^18,19^ and Ramanah et al.^20^. In patients without previous hysterectomy, the first step of the intervention consisted in executing complementary surgical procedures such as subtotal or total hysterectomy with or without salpingo-oophorectomy. Secondly, the sacral promontory was identified. Then, the peritoneum overlying the sacrum was opened to expose the sacral anterior longitudinal ligament. The peritoneal incision was caudally extended towards the pouch of Douglas. Using a vaginal manipulator, the rectovaginal septum was dissected to expose the levator ani muscles. Similarly, anteriorly, the vesico-vaginal space was dissected towards the bladder neck using the urinary catheter balloon as a landmark. The anterior and posterior polypropylene meshes were sutured to the levator ani muscles and to the vaginal walls using non-absorbable sutures while the surgeon’s fingers verified the absence of a point transfixing the vagina. Meshes were fixed on the cervix with sutures if present. One or both tails of the mesh were suspended to the anterior sacral ligament by two permanent sutures. Meshes were peritonized to avoid digestive loop incarceration.

Postoperative care was standardized across surgeons. In the absence of any complication, the urinary catheter was removed, and the patient was allowed to ambulate the day after surgery.

Patients were identified from data collected by the Medical Information Systems Program. Information was extracted from paper and electronic hospital medical records. Demographic and medical data as well as histories were collected during preoperative work-up. A physical examination was performed to determine pelvic floor disorders. POP was classified according to the POP-Q classification. Significant intraoperative complications (organ injuries or haemorrhage), early postoperative complications (haemorrhage, infection or early reoperation before hospital discharge) or late postoperative complications (reoperation after hospital discharge and before postoperative consultation) were recorded. All patients underwent a physical examination between six and eight weeks after surgery. Follow-up was performed once a year or more frequently, especially in case of persistent or new symptoms related to the POP surgery or failed surgery or recurrent prolapse. Thereafter, patients were seen again in case of new or persistent symptoms or recurrence. POP recurrence has been defined by a POP-Q stage ≥ 2 in at least one vaginal compartment^21–23^. Repeat surgeries for POP recurrence were performed based on functional and anatomical outcomes, not on isolated anatomical outcomes.

The primary outcome was reoperation procedures for a symptomatic recurrent POP. Secondary outcomes included operative time (time for midurethral sling placement not included), intraoperative organ trauma (bladder, bowel, vagina), intraoperative blood loss, postoperative POP recurrence (operated on or not), global reoperation and for mesh complications. Global reoperation included repeat surgeries for mesh complication or POP recurrence.

Quantitative variables were expressed as mean ± standard deviation and qualitative variables as number of cases (percentage). Qualitative data were compared using the Chi-square test or the Fisher’s exact test for small datasets. According to their distributions, quantitative variables were analyzed using the Student's t-test or Wilcoxon test. Statistical testing was carried out at the two-tailed level of 0.05. Data were analyzed with R 3.5 ® statistical software. No power calculation was performed because it is an exploratory study comparing first versus second line sacrocolpopexies in terms of morbidity and mid-term efficacy. This relation has never been explored before. But all patients from January 2012 to June 2019 were included.

### Ethic approvals

The study protocol was approved by the Institutional Review Board of the French College of Obstetricians and Gynecologists (CEROG 2019-GYN-0203) and the Dutch local Institutional Review Board (no. 088-708 34 87).

## Results

**Figure 1:**
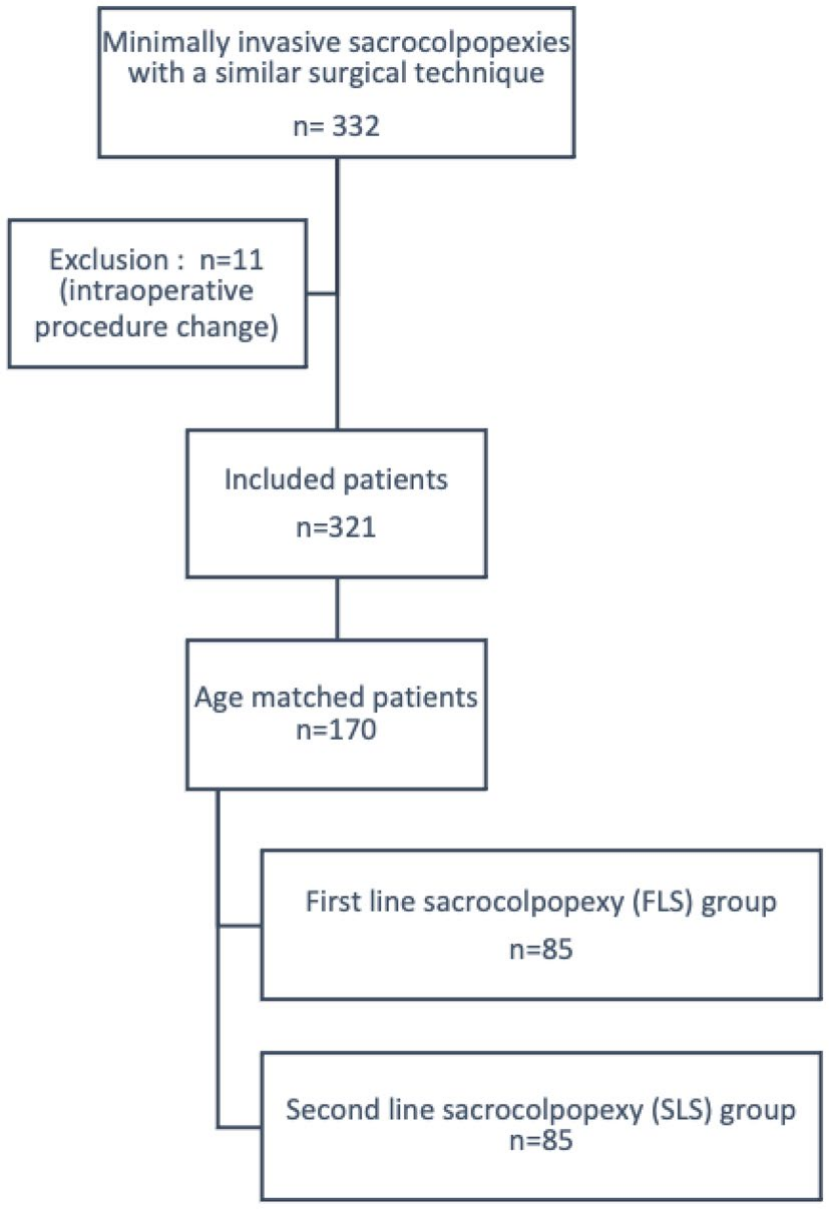
Flow chart.

During the period study, 332 patients underwent a laparoscopic or robotic sacrocolpopexy with a similar surgical technique. Among them, 11 patients were excluded because of procedural changes during surgery: 10 for an inaccessible promontory and 1 for a rectal trauma requiring a bowel resection. After age-matching, 170 patients constituted the study population: 85 patients in the FLS and SLS groups, respectively (Fig. 1).

The two groups were comparable in terms of demographics, clinical examination, and surgery management characteristics (Table 1). Patients from the SLS group had a higher rate of history of total or supracervical hysterectomy (79% versus 3.5%, p < 0.0001). Prior hysterectomy was performed for gynecological reasons and not for prolapse. Patients from the SLS group also had a lower rate of laparoscopic sacrocolpopexies (25.9% versus 75.3%, p < 0.0001) and all had a history of vaginal surgery for POP. All patients had at least a stage II POP of at least one vaginal compartment. Operative times were not statistically different between the two groups (198 ± 67 min versus 193 ± 60 min, p = 0.5). Only ten patients (100% in the FLS group) underwent a concomitant stress urinary incontinence surgery with mid-urethral sling. No Burch urethropexy was performed.

**Table 1:** Comparison of demographic, history, clinical examination, and surgericaly management characteristics between first line and second line sacrocolpopexies.

n = 170	First line sacrocolpopexy (n = 85)	Second line sacrocolpopexy (n = 85)	p-value
Demographic characteristics and history			
Age (years)	58.4 ± 8	58.7 ± 8	0.8
Body mass index (kg/m^2^)	25.6 ± 4.2	26.8 ± 3.8	0.09
Parity	2.5 ± 1.2	2.6 ± 1.1	0.4
Menopause	62 (73)	72 (85)	0.06
History of total or supracervical hysterectomy	3 (3.5)	67 (79)	** < 0.0001**
History of urinary incontinence surgery	3 (3.5)	1 (1)	0.6
Type of first surgery			
Sacrospinous ligament fixation	–	5 (6)	
Anterior Colporrhaphy	–	48 (56.5)	
Posterior Colporrhaphy	–	48 (56.5)	
Manchester surgery	–	9 (10.6)	
Clinical examination			
POP-Q Ba point (cm)	0.8 ± 2	0.6 ± 1.8	0.6
POP-Q Apical point (C or D based on previous total hysterectomy) (cm)	-0.6 ± 3	-1.6 ± 3.2	0.2
POP-Q Bp point (cm)	-1.6 ± 1.9	-0.9 ± 1.5	0.1
TVL (cm)	9.2 ± 1.7	8.7 ± 1.5	0.2
Operative management			
Laparoscopic approach	64 (75.3)	22 (25.9)	** < 0.0001**
Operation duration (minutes)	198 ± 67	193 ± 60	0.5
Type of concomitant hysterectomy	82 (96.5)	18 (21.2)	0.1
Supracervical hysterectomy	80 (94.1)	16 (18.8)	
Total hysterectomy	2 (2.4)	2 (2.4)	
Anterior and posterior meshes	80 (94.1)	85 (100)	0.06
Mean follow-up time (months)	38 ± 25	33 ± 25	0.2

Values are mean ± deviation standard or number of cases (percentage).

*n* number of cases, *cm* centimeters, *POP-Q* pelvic organ prolapse quantification system, *TVL* total vaginal length.

Significant values are in bold.

The mean follow-up time was similar in both groups: 38 ± 25 months in the FLS group and 33 ± 25 months in the SLS group (p = 0.2). Only six patients (3.5%) defaulted scheduled follow-up. All belonged to the FLS group. There was no statistically significant difference between the two groups in terms of recurrent POP operated or not (9.4% versus 10.6%, p = 0.7), repeat surgery for recurrent POP (3.5% versus 5.9% p = 0.7), mesh-related reoperation (0% versus 2.4%, p = 0.5) and global reoperation (3.5 versus 8.2%, p = 0.3) (Table 2). POP recurrence was diagnosed significantly earlier in FLS patients (4.6 ± 4.6 months versus 28.8 ± 15.6 months, p = 0.01). There was statistically no association between the age or the type of surgical approach (laparoscopic or robotic) and prolapse recurrence reoperated or not (p > 0.05). Repeat surgery for POP recurrence was not for residual prolapse but for real recurrence in the 2 groups. Their physical examination were normal between six and eight weeks after surgery.

**Table 2:** Comparison of post-operative complications during follow-up between first line and second line sacrocolpopexies.

	First line sacrocolpopexy (n = 85)	Second line sacrocolpopexy (n = 85)	P-value
Recurrent POP (POP-Q ≥ II)	8 (9.4)	9 (10.6)	0.7
POP-Q point			
POP-Q Ba point (cm)	0.7 ± 1.5	0.4 ± 1.5	0.8
POP-Q Apical point (C or D based on previous total hysterectomy) (cm)	− 3 ± 4	− 0.5 ± 0.6	0.1
POP-Q Bp point (cm)	− 0.6 ± 1.7	0.6 ± 1.3	0.2
Age (years)			0.7
< 40	0	0	
40–49	0	1 (11.1)	
50–59	5 (62.5)	3 (33.3)	
60–69	3 (37.5)	4 (44.4)	
70–79	0	1 (11.1)	
> 80	0	0	
Global reoperation	3 (3.5)	7 (8.2)	0.3
Recurrent POP reoperation	3 (3.5)	5 (5.9)	0.7
Age (years)			1
< 40	0	0	
40–49	0	0	
50–59	1 (33.3)	2 (40)	
60–69	2 (66.6)	3 (60)	
70–79	0	0	
> 80	0	0	
Vaginal compartment indicating POP reoperation			1
≥ 1 same compartment as the POP initial surgery	3 (100)	5 (100)	
≥ 1 another compartment than the initial POP surgery	0	1 (20)	
Type of surgery			1
Laparoscopic approach	1 (33.3)	3 (60)	
Robotic approach	2 (66.7)	2 (40)	
Mesh-related reoperation	0	2 (2.4)	0.5
Wound herniation	0	1 (1.2)	
Intestinal perforation	0	1 (1.2)	

Values are mean ± deviation standard or number of cases (percentage).

*n* number of cases, *cm* centimeters, *POP* pelvic organ prolapse, *POP-Q* pelvic organ prolapse quantification system.

There was no statistically significant difference between the two groups in terms of intraoperative complications such as organ injury (4.7% versus 7.1%, p = 0.7) and blood loss > 500 mL (2.4% versus 0%, p = 0.5) (Table 3).

**Table 3:** Comparison of intraoperative complications between first line and second line sacrocolpopexies.

	First line sacrocolpopexy (n = 85)	Second line sacrocolpopexy (n = 85)	p-value
At least one event	4 (4.7)	6 (7.1)	0.7
Vaginal perforation	2 (2.4)	1 (1.2)	
Bladder perforation	1 (1.2)	3 (3.5)	
Intestinal perforation	1 (1.2) 0 (2.4)	1 (1.2) 2 (2.4)	
Gastric perforation	1 (1.2)	0	
Bleeding from trocar opening	0	1 (1.2)	
Vascular lesion	0	0	
Blood loss ≥ 500 mL	2 (2.4)	0	0.5

Values are mean ± deviation standard or number of cases (percentage).

*n* number of cases, *mL* milliliters.

## Discussion

In our study, morbidity and recurrent POP rate at mid-term follow-up (3 years) seemed similar after first line and second line sacrocolpopexies. A prior prolapse repair appeared not to be a risk factor for increased operative time (198 min versus 193 min, p = 0.5), intraoperative surgical complications (4.7% versus 7.1%, p = 0.7), mesh related reoperations (0% versus 1.2%, p = 0.5). We found no significant difference between FLS and SLS groups in terms of prolapse recurrence rates, reoperated (3.5% versus 5.9%, p = 0.7) or not (9.4 versus 10.6, p = 0.7). In line with our results, a recent study published by Dubinskaya et al. also reported no difference (age adjusted) in terms of operative time (7.78 min shorter in case of previous prolapse repair, p = 0.27), major complication (15.8% versus 16.1%, p = 0.914) and mesh-complication (4.4% versus 4.2% p = 1) between 641 and 174 patients who underwent a laparoscopic or robotic sacrocolpopexy without and with a history of prior prolapse repair respectively^24^. In their study, prolapse recurrence was not affected by prior prolapse repair (aOR = 0.94 CI 95% [0.53–1.67]). In a prior study with another surgical approach, it has been shown that outcomes were similar between patients who had prior prolapse repair and those who underwent their index prolapse repair surgery^25^.

To draw a final conclusion on the POP recurrence rate between the two groups, the follow-up duration is of utmost importance. Patients in the FLS and SLS groups had a similar mean follow up of 38 months and 33 months (p = 0.2). This mid-term postoperative follow-up could impact our ability to diagnose prolapse recurrence. However this rate is probably low. According to Cullingan et al., 2 years follow up after an abdominal sacrocolpopexy is reasonable because in their study, prolapse recurrence occurred within 6 months (70%), 1 year (81%) and within 2 years after surgery (95%)^26^.

We found a significant difference in the duration of diagnosis a POP recurrence. In the FLS group, recurrence was diagnosed 24 months earlier as compared to SLS group (4.6 ± 4.6 months versus 28.8 ± 15.6 months, p = 0.01). There are two possible reasons for this. The first one could be that patients who had already undergone two prolapse surgeries and were aware of the possible limitations of POP surgery, took more time to seek medical assistance or tried a conservative approach such as the use of pessaries before consulting when they experienced symptoms suggesting another prolapse recurrence. The second reason could be that recurrence actually occurred later after double POP surgery.

POP recurrence was the first indication for reoperation in both groups (3.5% versus 5.9%, p = 0.7). A similar rate was found by Vandendriessche et al. who noted a reoperation rate for recurrent POP of 5.1% after 22 months in a retrospective study of 464 patients who underwent a laparoscopic sacrocolpopexy including 13.6% of patient with a history of previous prolapse repair^6^.

In a recent study published by Rebahie et al., the existence of variations in surgical practices between physicians was reported^27^. In order to control differences between the sacrocolpopexy techniques among the centers in our study, we set the following limitations: no sacrohysteropexy and only sacrocolpopexies performed with the same technique according to the operative reports were included (dissection of the vagina anteriorly towards the bladder neck only, fixation on the levator ani muscle only if concerned and mesh attachment on the promontory with knots). The points of divergence in practices within the community of urogynecologic surgeons were essentially the indication for a concomitant hysterectomy or a suburethral sling or a posterior mesh^27^.

Patients were age-matched in order to improve strength and comparison between the groups^21^. Data were collected from direct review of medical records, operative reports, office and emergency appointment by physicians from 3 European departments experts in urogynecology. Its important external validity makes these results more applicable to the general population.

The main limitation of this study is its retrospective design and the lack of randomization. Therefore, this study lacks an objective and functional assessment of pelvic floor symptoms. Prolapse quality of life questionnaires were introduced a few years ago in our reference centers and concerned only a small part of the study population. In any case, all patients underwent a surgery because they were symptomatic (e.g. urinary or bowel symptoms, deterioration of activity, difficulties in social relationships and self-image). POP surgeries and recurrent POP surgeries were performed based on functional and anatomical outcomes.

Another limitation concerns lost patients whose complications or reoperations could not be assessed. However, we consider this rate to be low and non-significant because both hospitals involved in this study are Regional Pelvic Floor Referral Centers.

In this study, the number of included patients over 75 years old is restricted. Initially, we could include 321 patients. After age matching, the number of patients was reduced because of the limited number of older patients who underwent a first line sacrocolpopexy. This bias probably did not impact our results. A study published by Boudy et al. demonstrated that perioperative complications and reoperation rates were similar during laparoscopic sacropexy among 191 patients over and under 70 years of age^28^.

## Conclusion

Patients who underwent a first or a second line sacrocolpopexy seemed to have similar rates of prolapse recurrence, major complication, mesh-related complications and operative times. Nevertheless, further prospective comparisons of perioperative and long-term outcomes of recurrent POP between first and second lines sacrocolpopexies in larger prospective studies are needed.

## Data Availability

The datasets generated and/or analysed during the current study are not publicly available due to the absence of consent from all patients for publication of their data but are available from the corresponding author on reasonable request.
